# Co-Administration of Simvastatin Does Not Potentiate the Benefit of Gene Therapy in the mdx Mouse Model for Duchenne Muscular Dystrophy

**DOI:** 10.3390/ijms23042016

**Published:** 2022-02-11

**Authors:** Nathalie Bourg, Ai Vu Hong, William Lostal, Abbass Jaber, Nicolas Guerchet, Guillaume Tanniou, Fanny Bordier, Emilie Bertil-Froidevaux, Christophe Georger, Nathalie Daniele, Isabelle Richard, David Israeli

**Affiliations:** 1 Généthon, 91000 Evry, France; bourg@genethon.fr (N.B.); avuhong@genethon.fr (A.V.H.); wlostal@genosafe.com (W.L.); ajaber@genethon.fr (A.J.); guerchet@genethon.fr (N.G.); gtanniou@genethon.fr (G.T.); fbordier@genethon.fr (F.B.); ebertil@genethon.fr (E.B.-F.); georger@genethon.fr (C.G.); daniele@genethon.fr (N.D.); richard@genethon.fr (I.R.); 2 Université Paris-Saclay, Univ Evry, Inserm, Genethon, Integrare Research Unit UMR_S951, 91000 Evry, France

**Keywords:** Duchenne muscular dystrophy, simvastatin, gene therapy, AAV, microdystrophin, combined therapy, lipid metabolism

## Abstract

Duchenne muscular dystrophy (DMD) is the most common and cureless muscle pediatric genetic disease, which is caused by the lack or the drastically reduced expression of dystrophin. Experimental therapeutic approaches for DMD have been mainly focused in recent years on attempts to restore the expression of dystrophin. While significant progress was achieved, the therapeutic benefit of treated patients is still unsatisfactory. Efficiency in gene therapy for DMD is hampered not only by incompletely resolved technical issues, but likely also due to the progressive nature of DMD. It is indeed suspected that some of the secondary pathologies, which are evolving over time in DMD patients, are not fully corrected by the restoration of dystrophin expression. We recently identified perturbations of the mevalonate pathway and of cholesterol metabolism in DMD patients. Taking advantage of the mdx model for DMD, we then demonstrated that some of these perturbations are improved by treatment with the cholesterol-lowering drug, simvastatin. In the present investigation, we tested whether the combination of the restoration of dystrophin expression with simvastatin treatment could have an additive beneficial effect in the mdx model. We confirmed the positive effects of microdystrophin, and of simvastatin, when administrated separately, but detected no additive effect by their combination. Thus, the present study does not support an additive beneficial effect by combining dystrophin restoration with a metabolic normalization by simvastatin.

## 1. Introduction

Duchenne muscular dystrophy (DMD) is the most common inherited pediatric muscle disorder. It is an X-linked genetic progressive myopathy characterized by muscle wasting and weakness, which leads to loss of motor functions, cardiac and respiratory impairment, and premature death [1]. DMD occurs at a rate of approximately 1:5000 male births and arises due to mutations in the dystrophin gene. The disease is caused by a deficiency of functional dystrophin, a critical component of the dystrophin-associated protein complex that links the cytoskeleton with the extracellular matrix in skeletal and cardiac muscles [2]. The primary direct consequence of the disruption of this linkage by the lack of dystrophin is thought to involve sarcolemma destabilization, perturbation of Ca^2 +^ homeostasis, activation of proteases, mitochondrial damage and tissue degeneration. The only routinely used medication for DMD patients is glucosteroid drugs, which can at best only slightly delay the disease progression [3], however, gene correction and gene replacement technologies have emerged in recent years as promising treatment options for DMD.

Such experimental therapeutic approaches, based on gene therapy, cell therapy and drug discovery, are focused on the restoration of dystrophin expression [4,5,6,7]. Despite increasing efficiency in the restoration of dystrophin expression, muscle functional improvement in clinical trials is yet unsatisfactory. A likely simple explanation for this limited therapeutic efficacy is the technical inability to express at sufficient level (micro) dystrophin at the right time and place. An alternative but non-mutually exclusive explanation for the only modest functional improvement in clinical trials is that perhaps the restored dystrophin cannot completely reverse some of the pathological manifestations in the dystrophic muscle. Indeed, the patients’ dystrophic tissues present evidence for a complex array of pathological changes, including myofiber degeneration/regeneration, increased sarcolemma Ca^2 +^ level, perturbed nNOS signaling, TGF beta signaling and fibrosis, energy metabolism dysregulation, lipid accumulation, calcification, necrosis, and inflammation [1,8,9]. It is reasonable to hypothesize that some of these pathological changes cannot be completely reversed by the sole restoration of dystrophin. The challenge is therefore to identify the most critical secondary pathologies in DMD and to address their reversion, in order to propose complementary treatment. Such treatment may then be complementary to the restoration of dystrophin by gene therapy, in the setup of a combined therapy approach.

In 2015, the Froehner’s group demonstrated a substantial improvement of the functional parameters of skeletal muscles in the mdx mouse that was treated by the cholesterol-lowering drug, Simvastatin [10]. This improvement of muscle function was however thought to be cholesterol-independent, but to involve pleiotropic effects of simvastatin [11], including reduction of oxidative stress, inflammation and fibrosis, all of which being deleterious pathways known to contribute to the pathogenesis of DMD [9]. A 2019 follow-up study of the same group demonstrated that simvastatin markedly improves cardiac functions in the dystrophic mdx mouse, and therefore may provide a novel approach for treating cardiomyopathy in DMD [12]. In the general population, however, the use of statin is well known to be associated with a risk for the development of deleterious muscle side-effects [13]. This makes the use of statin contra-intuitive, in the context of muscular dystrophy. In this context, the attempts to reproduce these promising results by other independent laboratories had failed, since the treated mdx mice did not present muscle functional improvement [14,15]. These negative results are explained possibly by the relatively low level of simvastatin that was measured in the treated mice [16], yet, the failure to validate the early results, associated with the statins’ notorious propensity to cause muscle pain in the general population [13], raised doubt on the therapeutic hopes of simvastatin administration in DMD.

In a recent study, we profiled circulating miRNAs in a large cohort of DMD patients. We developed a new approach for the interpretation of miRNA dysregulation, which predicted that the mevalonate pathway and cholesterol metabolism are dysregulated in DMD [17]. Treating mdx mice with simvastatin during a short period of only three weeks resulted in reduced diaphragmatic fibrosis and reduced level of muscle damage biomarkers, muscle creatine kinase (mCK) and myomesin−3 (Myom−3). Our results supported that the canonical activity of simvastatin consisting of the inhibition of HMGCR activity and cholesterol synthesis, are the direct mediators of the simvastatin effect in the dystrophic muscle. In addition to shedding light on the mechanism of the positive effect of simvastatin, these results supported that simvastatin could be an attractive drug for a combined approach in DMD. In the present study, we therefore tested, in the mdx mouse model, the feasibility of a therapeutic approach based on the combination of the restoration of dystrophin expression, by a classical gene-therapy approach, with a metabolic normalization treatment by a treatment with Simvastatin.

## 2. Results

### 2.1. Experimental Design and Microdystrophin Expression

**Figure 1:**
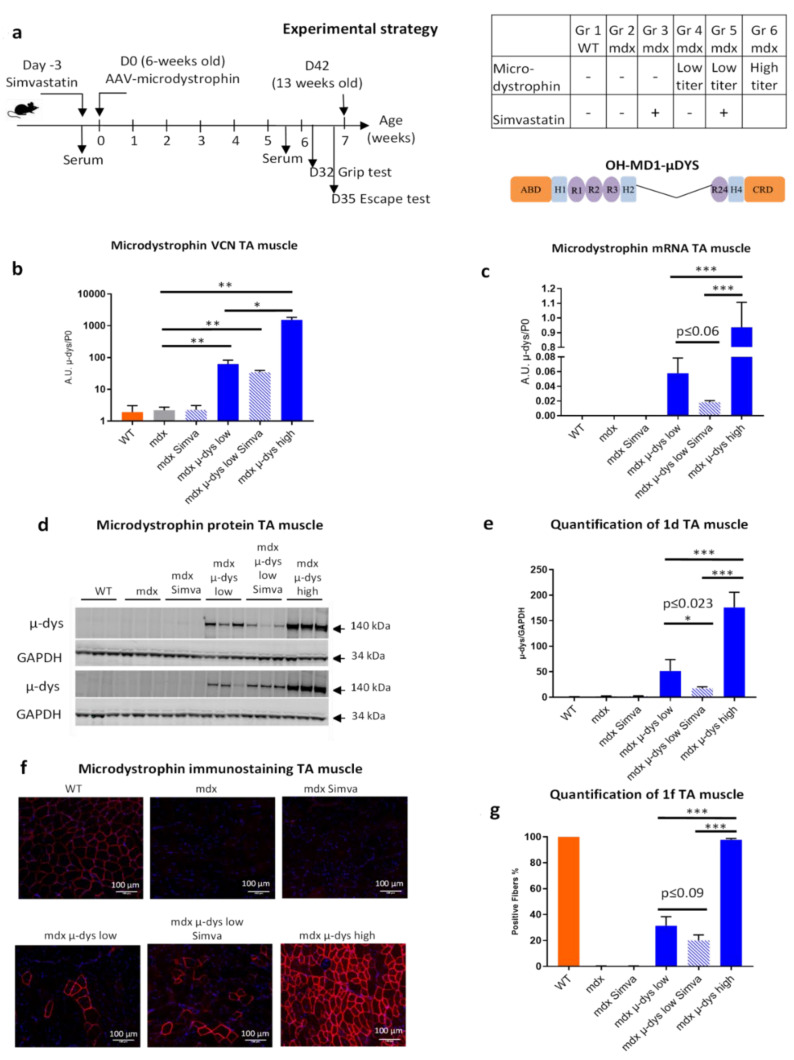
Experimental design and transgene expression. (**a**) Young adult mdx mice (*n* = 6) and their matched healthy controls were treated in the experimental groups as reported in the table, for the duration of seven weeks. Simvastatin treatment (orally 80 mg/kg) started three days before transgene application. The AAV9 optimized human microdystrophin vector (a µdysΔ4−23ΔCT construct (designated OH-MD1-µDys), under the transcriptional control of the artificial Spc5.12 promoter) was administrated intravenously by tail vein injection. OH-MD1-µDys is composed of actin-binding domain (ABD), hinge regions (H1, 2, 4), spectrin-like repeat regions (R1, 2, 3, 24), and the cysteine-reach domain (CRD), which mediates binding to the dystroglycoprotein complex. (**b**) Vector copy number (VCN), normalized to the genomic *Rplp0* (P0) was evaluated in the TA muscle. (**c**) mRNA expression of the microdystrophin (µ-dys) in the TA muscles was quantified by RT-qPCR. (**d**) The expression of the microdystrophin in the TA muscles was quantified by a Western blotting (*n* = 6) and presented graphically in (**e**) after normalization to GAPDH. (**f**). Transversal sections of the TA muscles were stained for dystrophin expression. (**g**). Quantification and graphical presentation of **f**, Scale bar = 100 µm. * *p* < 0.05; ** *p* < 0.01, *** *p* < 0.005.

In the present study, we evaluated a combined therapy approach in a DMD mouse model, by adding treatment of the anti-cholesterol drug simvastatin with the restoration of dystrophin expression by the intravenous delivery of an AAV-microdystrophin. Young adult (six weeks old) mdx were randomly assigned to five treatment groups (*n* = 6), as compared to a healthy C57Bl/10 control group. The comparison groups included: (1) control healthy C57Bl/6 (WT), (2) untreated mdx (mdx), (3) mdx treated with Simvastatin only (mdx Simva), (4) mdx treated with AAV-microdystrophin at low titer (mdx µ-dys-low), (5) mdx treated with AAV-microdystrophin at low titer plus simvastatin (mdx µ-dys-low + simva), and (6) mdx treated with AAV-microdystrophin at high titer (mdx µ-dys-high) (Figure 1a).

The viral titer that was selected for the high titer group (the 6th group) of 3 × 10^13^ viral genome (vg)/kg, which is, in our experience, required to transduce close to 100% of the myofibers in the TA and the GA muscles in the young adult mdx. The low titer that was selected (the 4th group), of 5.0 × 10^12^ vg/kg, (a 1/6 of the high titer), is the dose that in our knowledge and experience required for the transduction of below 25% of the myofibers of the TA and GA muscles in mdx, which is below the threshold for a measurable muscle function benefit. This experimental design was made in order to test whether the combination of a low dose with a boosting drug (simvastatin here) (in the 5th group) could improve the dystrophic tissue and/or provide functional benefit to the dystrophic muscle to a level, close to the high titer (6th group) treated group. Simvastatin was mixed into a standard rodent diet at a concentration of 80 mg/kg, which is a dose within the normal range prescribed for humans, based on mouse-to-human equivalence calculations, and that demonstrated therapeutic efficacy in the mdx model [10], improved diaphragm fibrosis and reduced levels of circulating biomarkers for muscle damage [17]. Simvastatin treatment started three days before (D−3) the administration of a recombinant adeno-associated virus expressing the human codon-optimized microdystrophin (µ-dys) cDNA under the control of the Spc5.12 promoter, using the intravenous delivery of AAV9 serotype (rAAV9-Spc5.12-hum-microdystrophin), which is characterized by high affinity for the skeletal muscle [18]. Muscle functional tests were performed between the six and seven weeks after the treatment until the experimental endpoint at 13 weeks of age (Figure 1a). At the endpoint of the experiment, the expression level of the AAV recombinant vector was monitored by RT-qPCR, using specific human dystrophin primers.

Vector copy-number (VCN) quantification (normalized to murine nuclear genomic DNA—*Rplp0*) in the Tibialis Anterior (TA), muscle presented a proportionally dose-responsive increased expression from AAV-untreated, to the low and high-dose AAV injected animals, (Figure 1b). Similar levels of the viral genome between the mdx µ-dys-low and mdx µ-dys-low + Simva groups indicated no detectable effect of simvastatin on the viral vector’s infectivity of the muscle tissue. A similar pattern of vector integration was detected in the Gastrocnemius (GA) and the diaphragm muscles, here too, without a detectable effect of simvastatin treatment (Supplementary Figure S1a,b).

An RT-qPCR analysis was performed for the detection of the human microdystrophin mRNA in the TA muscle of the injected mice and detected a dose-dependent increased expression in the low and high-titer mdx groups. Microdystrophin mRNA level in the combined therapy group (µ-dys-low + Simva) was reduced, on the limit of significance, compared to µ-dys-low treatment alone (Figure 1c, *p* = 0.06). A small reduction, of microdystrophin mRNA level in the simvastatin treated group (µ-dys-low + Simva compared to µ-dys-low alone) was detected also in the GA muscle (*p* ≤ 0.072), but not in the diaphragm, where simvastatin treatment did not affect the mRNA level of the µ-dystrophin transgene (Supplementary Figure S1c,d).

In agreement, a Western blotting analysis for the detection of the 140 kDa human microdystrophin protein in the same (TA) muscles detected a dose-dependent increased expression from the low to the high-titer mdx groups, and a reduced microdystrophin protein level in the combined therapy group (mdx µ-dys-low + Simva) compared to µ-dys-low treatment alone (Figure 1d,e, *p* ≤ 0.023, one-way ANOVA). However, such reduction (mdx µ-dys-low + Simva compared to µ-dys-low, treatment alone) was not detected in the GA muscle (Supplementary Figure S1e,f). For the immune-fluorescence detection of dystrophin positive fibers, we used the DYSB antibody, raised against the N-terminus human dystrophin, and detected to a lower level also the mouse dystrophin. The dystrophin staining of transversal sections TA muscle thus detected a low signal of endogenous mouse dystrophin in the WT healthy mouse (Figure 1f) as compared to the high signal of the ectopically-expressed human microdystrophin transgene. Only a small number of revertant fibers was detected in the non-injected mdx mice, and no significant effect of simvastatin was detected on this background level (Figure 1f,g). Mdx µ-dys-high muscles showed close to 100% positive fibers (Figure 1f,g). In the lower-dose treatment of microdystrophin, TA muscle from mdx µ-dys-low had in average 31 ± 6.95% positive fibers, whereas a lower level, although not significant (*p* = 0.09, Student *t*-test), of 20 ± 4.19% dystrophin positive fibers was detected in the mdx µ-dys-low + Simva group (Figure 1g). A similar expression pattern was detected in the GA muscle, of which, however, only a small non-significant reduction of transgene expression was detected in the simvastatin-treated mice of the low titer microdystrophin (Supplementary Figure S1g,h). Taken together, this data suggested a normal dose-response of the microdystrophin viral vector in the mice that were not treated by simvastatin, while a tendency for reduced microdystrophin expression from the transgene was observed at both mRNA and protein levels in the simvastatin-treated mice.

### 2.2. Histological Evaluation

**Figure 2:**
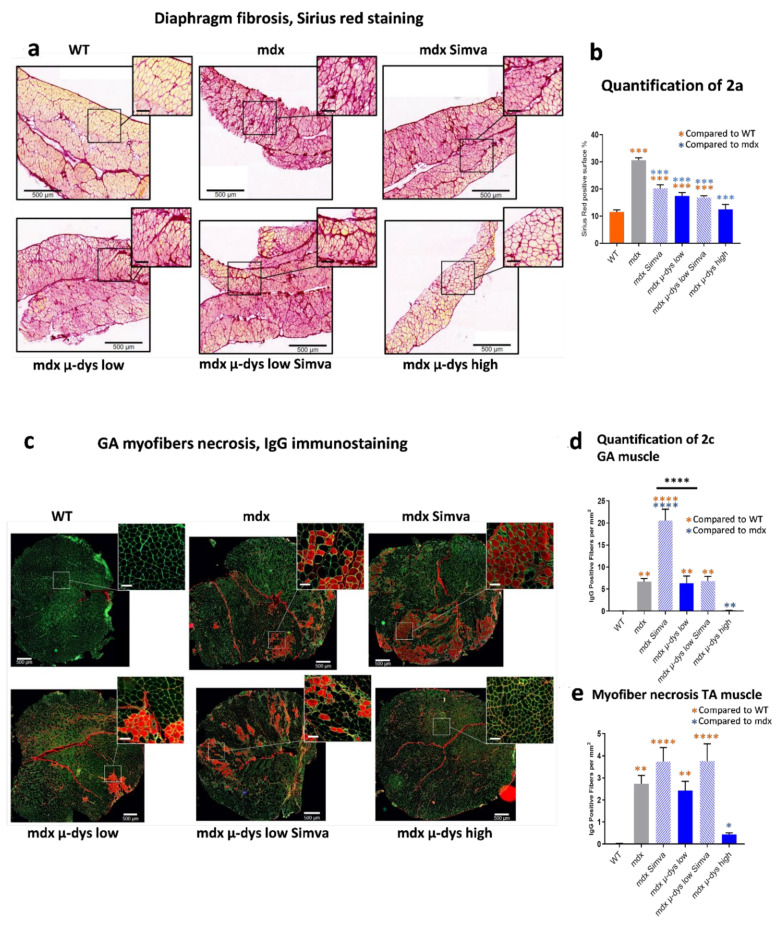
Histological characterization. (**a**) Representative images of fibrosis (Sirius red), transversal sections of the diaphragm muscle. (**b**) Quantification of 2a. (**c**). Representative images of IgG-positive necrotic fibers (red), co-stained with anti laminin antibody (green) in GA muscles. (**d**). Quantification and graphical presentation of (**c**). (**e**). Quantification and graphical presentation of IgG-positive myofibers in the TA muscles (images of the TA muscles are not shown). Scale bars of (**a**,**c**) = 500 µm in regular panels and 100 µm in zoom-in inserts. * *p* < 0.05; ** *p* < 0.01, *** *p* < 0.005, **** *p* < 0.001.

Analysis of muscle fibrosis: In the groups without simvastatin treatment, fibrosis levels in diaphragm muscle (quantified by Sirius Red positive area) was elevated in untreated mdx compared to the wild-type and presented an expected dose-responsive effect with AAV-microdystrophin treatment: mdx µ-dys-low only partially restored fibrosis staining while mdx µ-dys-high brought it back to the wild-type level (Figure 2a,b). Previous studies demonstrated reduced fibrosis levels in simvastatin-treated mdx mice [10,17]. Similarly, we detected here a significantly reduced Sirius-red staining level in mdx mice treated by simvastatin only (Figure 2a,b). Low titer microdystrophin and the combined low microdystrophin + simvastatin treatments reduced fibrosis to a similar level, slightly below simvastatin only, but yet still significantly higher than the control WT group. Only the high titer microdystrophin treatment reduced the Sirius-red level to that of the control WT group (Figure 2a,b).

Analysis of myofiber damage was assessed by IgG staining, which is nonspecifically attached to necrotic myofibers [19]. In agreement with [14], we detected a significantly increased level of necrotic myofibers in the GA muscle of the mdx simvastatin group, as compared to the untreated mdx mice. In contrast, no effect of simvastatin on GA muscle myofiber necrosis level was detected in mice that were treated by the low titer microdystrophin (Figure 2c,d). Overall necrosis rate was reduced in the TA compared to the GA muscle (images not shown). In the TA muscle simvastatin treatment increased slightly necrosis rate, below significance level, in both untransduced mdx mice and in the low titer microdystrophin treated mice (Figure 2e).

### 2.3. Evaluation of Circulating Biomarkers and Muscle Functional Examination

**Figure 3:**
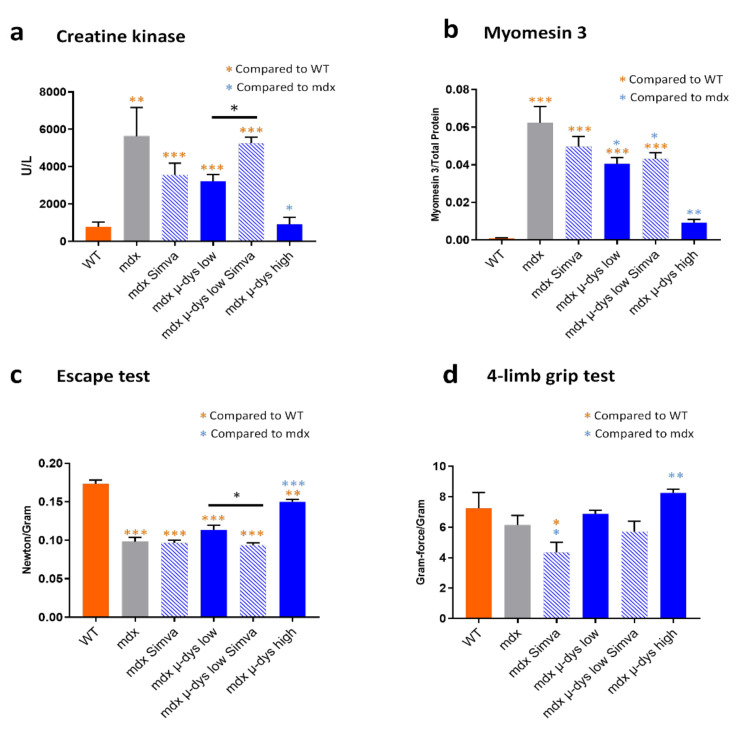
Circulating biomarker and muscle functional assessment. (**a**,**b**). Serum mCK and myomesin−3 quantifications, respectively. (**c**) Results of the “escape test”, for the evaluation of whole-body force generation, normalized to body mass. (**d**) Results of the 4-limbs grip force, normalized to body mass. * *p* < 0.05; ** *p* < 0.01, *** *p* < 0.005.

In agreement with Amor et al. [17], simvastatin treatment reduced mCK values as compared to the untreated mdx group (Figure 3a), which however did not reach significance. In the mdx group injected by the low titer microdystrophin, the mCK level was reduced, again, not reaching significance compared to untreated mdx. Surprisingly, the combination of low microdystrophin + simvastatin resulted in increased mCK level, to a significantly higher level as compare to microdystrophin low only (*p* = 0.037) (Figure 3a). Finally, as expected, the microdystrophin high treatment reduced mCK to the level of the control healthy mouse, thus presenting the expected dose-response of mCK to the dystrophin level, in the groups that were not treated by simvastatin. The treatments of mdx mice by Simvastatin and by the low titer microdystrophin reduced slightly, not significantly, myom−3 level as compared to untreated mdx (Figure 3b). The treatment by simvastatin + low titer microdystrophin increased Myom−3 level slightly but not significantly, compared microdystrophin only. Myom−3 level was reduced drastically in the high titer microdystrophin treated mdx, to yet a higher level (but not significantly different) from the control healthy group. Whole-body force generation was measured by the escape test. The reduced level of the untreated mdx was significantly improved only by the high-titer AAV-microdystrophin treatment (Figure 3c), which, however, was still significantly below the level of the healthy control group. Interestingly, mdx mice that were treated by combined low titer microdystrophin and simvastatin presented significantly reduced force generation capacity compared to the low microdystrophin-only group. Grip capacity was reduced, however insignificantly, probably due to high variability, compared to the healthy control group (Figure 3d). High titer microdystrophin, but not low titer, rescue the grip capacity in the mdx mice, to a level similar to the healthy control group. Of interest, the treatment of mdx group by simvastatin only reduced the grip capacity to a level below the untreated mdx group.

## 3. Discussion

Recently, we found strong evidence for the perturbations of cholesterol metabolism in DMD [17]. In agreement with previous works [10,12], we found that in mdx mice the treatment by simvastatin improved some of the pathological parameters, among the reduction of diaphragm fibrosis and reduced serum levels of circulating biomarkers for myofiber damage, mCK and Myom−3. To explore the possible benefit of the combined approach, we designed a dose-escalating experimental system for the restoration of dystrophin expression in an AAV-microdystrophin gene therapy approach. We selected a low-dose viral titer slightly below the minimum expected level of dystrophin which is required for detectable improved muscle function in the mdx model, and a high-dose viral titer that transduced nearly all myofibers in the TA and GA muscles and thus produce a measurable muscle function improvement. This experimental design allowed testing whether a combination of low titer dystrophin restoration with simvastatin supplementation might provide a therapeutic benefit beyond that of the low titer of microdystrophin alone, or of the simvastatin only treatments, to a closer level to the high-titer microdystrophin treatment.

For simplicity, we may interpret the results in the order of (1), the effect of the microdystrophin only, followed by (2), the effect of simvastatin only, and finally (3), the effect of their co-administration. Accordingly, in (1), the mouse groups that were not subjected to simvastatin treatment (microdystrophin-only groups), as expected, VCN (Figure 1b) and dystrophin expression (Figure 1c–g) increased progressively from the untreated mdx, to the low- and high-dose treated mdx mice. A similar dose-response was also detected in the histological analysis (fibrosis detection on the diaphragm, Figure 2a,b), circulating biomarkers (creatine kinase, Figure 3a and myom−3 Figure 3b). Finally, similar results were also observed in the functional studies in Figure 3c,d). Overall, these results confirmed the robustness of our gene-therapy-based experimental system in the mdx model.

In (2), the analysis of the simvastatin-only group, we observed a reduced diaphragm fibrosis level (Figure 2a,b) and a trend toward reduced mCK and myom−3 levels (Figure 3a,b) in the simvastatin treated compared to untreated mdx group. The detection of necrotic fibers provided, however, seemingly contradicting results, because we detected (in agreement with [14]) increased necrosis rate by simvastatin treatment in both the GA and the TA muscles. No effect of simvastatin-only was observed in the escape test group, while a slight force drop was recorded in the limb grip test.

Lastly, it is of particular interest to interpret the effect of the combined therapy, by comparing the groups of low-dose microdystrophin with and without simvastatin (groups 4 and 5). Concerning microdystrophin expression: while no effect of simvastatin was observed on the infectivity (VCN) of the viral vectors, a small reduction in the expression of the microdystrophin was detected, consistently at the mRNA and protein levels, in the simvastatin treated group (Figure 1c–g). Concerning the anti-fibrotic effect of simvastatin: the reduction of fibrosis by the low-dose microdystrophin was not boosted by simvastatin co-administration. Concerning indicators of myofiber damage: increased myofiber permeability and necrosis were detected in the TA muscles after simvastatin co-administration. The levels of the muscle-damage biomarkers mCK and myom−3 increased after simvastatin co-administration, significantly for mCK (Figure 3a) and slightly, not significantly, for myom−3 (Figure 3b). Concerning the muscle functional tests, a reduction of muscle force by the co-administration of simvastatin was recorded in the functional tests, significant in the “escape” (Figure 3c) and slightly below significance in the 4-limbs grip test, (Figure 3d). Taken together, these results do not support an overall beneficial effect of the co-administration of simvastatin in the setup of combined therapy in the mdx mouse model for DMD.

Thus, when administrated alone, simvastatin treatment improved diaphragm fibrosis, which however was not improved by the co-administration of simvastatin on top of microdystrophin treatment. Similarly, the myofiber damage indicators mCK, Myom−3 and IgG positive fibers, did not improve or even get worst (mCK) by the co-administration of simvastatin on top of microdystrophin treatment.

Altogether, the data may suggest a mixed effect of simvastatin on the dystrophic parameters in the mdx model. On the one hand, consistently, simvastatin reduces diaphragm fibrosis. On the other hand, simvastatin might reduce slightly the expression of the dystrophin transgene (although this observation merits further confirmation), and simvastatin alone, or in combination with microdystrophin, seems to increases the proportion of necrotic myofiber. Consequently, the muscle force tests did not indicate functional improvement by the co-administration of simvastatin, on top of the beneficial effect of the microdystrophin alone. Thus, we concluded that the co-administration of simvastatin does not potentiate the benefit of gene therapy in the mdx mouse model for Duchenne muscular dystrophy.

Study limitations: The Simvastatin treatment regime of the present study is identical to the 2015 study of the Froehner group [10], and to our own recent study [17], both of which validated positive simvastatin effects on diaphragm fibrosis and on the levels of circulating biomarkers in the mdx model. In 2020, two research groups concluded that simvastatin did not ameliorate disease pathology in mdx mice [15]. Similarly, Mucha and colleagues [14] were not able to confirm the positive effect of simvastatin in the mdx model. In a rebuttal letter, Whitehead and coauthors [16] responded that in the Verhaart study [15] the plasma level of simvastatin was below a threshold for therapeutic efficacy. At the time that Verhaart’s paper had been published, the mouse experiment of the present study was ended, without having the possibility for a posterior monitoring of the level of the plasma Simvastatin. One limitation therefore of the present study is the lack of data concerning the plasma levels of simvastatin in the treated mice. It is however important to keep in mind, in this context, that similar to [10] and [17] the treatment of simvastatin resulted in the present study in the reduction of diaphragm fibrosis, and tendency for reduction in the levels of circulating biomarkers in the mdx group treated by simvastatin only. Surprisingly, simvastatin treatment resulted also in increased proportion of necrotic fibers, in the reduced expression of microdystrophin, and muscle capacity, in the mdx group that was treated by microdystrophin. Taken together, the data support strongly that simvastatin treatment reached effective level in the present study.

Another limitation of the present study is the lack of characterization of the molecular mechanism of the reduced expression of the microdystrophin transgene under the condition of simvastatin treatment. This surprising observation shall be the subject of future investigations.

In summary, the present investigation does not support the combination of simvastatin with the gene-therapy restoration of dystrophin. While the results of the present combined approach are disappointing, the rationale for the quest for efficient complementary treatment in DMD, remained solid. An ongoing study in our group is focused on other approaches for metabolic normalization in DMD, to be combined with the restoration of dystrophin expression.

## 4. Materials and Methods

### 4.1. In Vivo Mice Experiments

C57Bl10 (WT/BL10) and mdx (C57BL/10ScSn-Dmdmdx/J) mice were obtained from Charles River laboratories, Miserey, France. Mice were housed in an SPF barrier facility with a 12-h light/dark cycle, and were provided with food and water ad libitum. In this study, only male mice were used. For the in vivo studies, the operators who performed vector delivery, sample and tissue collection, and functional analyses were blinded to the treatment groups.

Simvastatin was mixed into a standard rodent diet (A04 diet, SAFE, 89290, Augy, France, https://safe-lab.com/safe_en/ accessed on 1 December 2021) at a concentration of 80 mg/kg. Seven week-old mice were treated for seven weeks. Muscle biopsies were dissected, snap-frozen in isopentane in liquid nitrogen, and processed for RNA, protein and histological analyses.

### 4.2. Generation of the Microdystrophin Construct

The Opt-human MD1 (OH-MD1-µDYS) construct is our optimized version of the microdystrophin that was developed by the laboratories of Jeffrey Chamberlain (H2mDys) [20] and George Dickson (MD1µDYS) [21] and used (the dog version) in Genethons’ preclinical investigation in the Golden retriever muscular dystrophy (GRMD) dog model [22]. It is a spectrin-like repeat 4 to 23 deleted, C-terminal truncated microdystrophin (mycrodysΔ4−23ΔCT). To obtain this version of mycrodysΔ4−23ΔCT, the MD1µDYS was modified further, including additional codon-optimization (for human), removal of CpG methylation sites, removal of antisense ORFs and of sense ORFs larger than 100 bp. The modified vector was designated OH-MD1-µDYS.

### 4.3. Generation and Titration of Recombinant AAV Vector

HEK293-T cells, cultured in suspension, were transfected with the three plasmids coding for the adenovirus helper proteins, the AAV Rep and Cap proteins, and the ITR-flanked transgene expression cassette. Three days after the transfection, cells were harvested, chemically lysed, treated with benzonase (Millipore, Guyancourt, France), and filtered. Viral capsids were purified by affinity chromatography, formulated in sterile PBS, and the vector stock was stored at −80 °C. Titers of AAV vector stock were determined by using quantitative real-time polymerase chain reaction (qPCR). Viral DNA was extracted using the MagNA Pure 96 DNA and viral NA small volume kit (Roche Diagnostics, Indianapolis, IN, USA) according to the manufacturer’s instructions. PCR was performed in ABI PRISM 7900 HT Sequence Detector with Absolute ROX mix (Taqman, Thermo Fisher Scientific, Waltham, MA, USA), using the ITR-specific primers, forward 5′-CTCCATCACTAGGGGTTCCTTG-3′, reverse 5′-GTAGATAAGTAGCATGGC-3′ and the probe 5′-TAGTTAATGATTAACCC-3′.

### 4.4. Vector Copy Number

DNA was extracted from transversal sections of the TA muscle, using the NucleoMag Pathogen kit (Macherey Nagel, Hœrdt, France). qPCR for the detection of vector number was performed in duplicates with the LightCycler 480 system (Roche, CH-4070, Basel, Switzerland), using the SYBER Green technology according to the manufacturer’s instruction, with the forward primer (5′-GATTGAGAAGCTGCTGGACC-3′) and the reverse primer (5′-TCTGGTGGTGTAGCTGGAAG-3′), producing a 191 bp band.

### 4.5. Microdystrophin Protein Detection by Western Blotting

Proteins were extracted with RIPA lysis buffer (Thermo Fisher Scientific, Les Ulis, France) complemented with Protease Inhibitor Cocktails (Roche, Basel, Switzerland) and Benzonase (Millipore, Guyancourt, France). Total protein was quantified with Pierce™ BCA Protein Assay (Thermo Fisher Scientific). Protein extracts were separated on 3–8% Tris-Acetate polyacrylamide gel (Thermo Fisher Scientific) and transferred to a nitrocellulose membrane using the iBlot2 Dry Blotting system (Thermo Fisher Scientific). Dystrophin expression was detected with a mix of Dys-B (Leica: NCL-DYSB) diluted 1:200, and Dys−2 (Leica: NCL-DYS2), diluted 1:50, and normalized to GAPDH intensity, using the Odyssey technology (Thermo Fisher Scientific) scanner and Image Studio Lite 4.0 software (LI-COR Biosciences, Lincoln, NE, USA).

### 4.6. Detection of Microdystrophin on Transversal Sections

Dissected tissues were frozen in isopentane in liquid nitrogen. Transverse 8µm cryosections of the TA muscle were air-dried and stored at −80 °C. Slices were rehydrated 5 min in PBS, fixed 5 min in 3.7% ice-cold methanol-free PFA, permeabilized in 0.1% Triton-X100 for 5 min, and blocked 30 min in 10% goat serum in PBS (blocking buffer) and additional 30 min in Mouse-on-Mouse IgG 1× Blocking Solution. Sections were incubated with 1:50 primary antibody (Leica: NCL-DYSB) diluted with PBS in 10% blocking buffer overnight at 4 °C. Samples were washed twice in PBS and incubated for 45 min at room temperature (RT) with Alexa-conjugated secondary antibodies (Goat-anti-Mouse IgG1k, Thermo Fisher Scientific A21125), 1:1000 in 10% blocking solution at a dark humid chamber. Samples were washed three times in PBS and mounted in DAPI Fluoromount-G (Southern Biotech, Birmingham, AL, USA ref. 0100-20). Images were digitalized using Axioscan Z1 slide scanner (Zeiss, Jena, Germany) under a Plan-Apochromat 10×/0.45 M27 dry objective (Zeiss, Jena, Germany) and using a digital CMOS camera ORCA-Flash4.0 (Hamamatsu, Japan). The size of a pixel is 0.65 µm. Tile scan images were reconstructed with ZEN software (Zeiss, Jena, Germany). Total and positive fibers were manually counted on four random images (900 × 900 pixels) per transversal section, using the ImageJ software. *p*-values were calculated by a one-way ANOVA.

### 4.7. Histology and Fibrosis Analysis

Sirius-red stained transversal sections were scanned with the AxioScan (Zeiss, Jena, Germany) 10 ×. The images were analyzed by morphometry with the HISTOLAB (91090 Lisses, France) software.

### 4.8. mCK Quantification

Blood samples were collected by retro-orbital bleeding and quickly centrifuged for 10 min at 8000 rpm. Sera were harvested and further centrifuged to completely remove cell contaminants. The sera were finally stored at −80 °C until measurement. Ten μL of mouse serum was used to colorimetrically measure creatine phosphokinase concentration by FUJI DRI-CHEM nx500 system (DMV Imaging).

### 4.9. Myomesin 3 Quantification

Sera protein concentration was measured with Pierce^TM^ BCA Protein Assay (Thermo Fisher Scientific, Les Ulis, France). Protein extracts were separated on 4–12% Bis-Tris polyacrylamide gel (Thermo Fisher Scientific), and transferred to a nitrocellulose membrane using the iBlot2 Dry Blotting system (Thermo Fisher Scientific) for 8 min 30 at 20 V. Total protein on blotted membranes were determined with the Revert 700 Total Protein (LI-COR Biosciences, Lincoln, NE) staining. Membranes were washed, blocked in Odyssey Blocking Buffer (LI-COR), 1 h, room temperature. Primary antibody (MYOM3 17692−1-AP, Proteintech 1/500 in 50% blocking buffer) was incubated overnight at 4 °C. After washings in 1× TBST (Tris/HCl (pH 7.5—20 mM)/NaCl (150 mM)/Tween 20 (0.1%)), the diluted secondary antibodies (1/1000 in 50% Odyssey blocking buffer) were incubated 1 h at room temperature. Membrane images were acquired with the Odyssey Infrared Imaging System. Band density was quantified using Image Studio Lite 4.0 software (LI-COR Biosciences, Lincoln, NE, USA) and normalized to total protein values.

### 4.10. Evaluation of Muscle Force

The motor capacity of the mice was evaluated using the escape and the 4-limb grip tests. The procedures follow the recommendations of TREAT NMD SOP for the DMD animal models [23]. Briefly, in the escape test, the mouse is placed on a platform inside a 30 cm tube, tail-connected to a force measurement devise, to record the escape force. A mean of the five highest scores out of max 15, during max 5 min (the first to be reached) is normalized to the weight of the mouse. The escape test is performed in only one session (i.e., the indicated number of repetitions in one single training session), since mice are unwilling to perform the escape test a second time. In the 4-limb grip strength, the grip force was measured by the grip strength meter, (Bioseb https://www.bioseblab.com/ accessed on 1 December 2021; France Grip Test 25N). Three independent measurements were performed, and the mean value of weight-normalized grip strength was calculated.

### 4.11. Data and Statistical Analysis

The Rstudio and GraphPad PRISM 7.01 program (GraphPad Software Inc. La Jolla, CA, USA) was used for statistics except for the data in Figure 3a,b. The results, which are presented in all the corresponding figures, represent the average ± SEM. ANOVA followed by Tukey HSD post-hoc test were used for multiple comparisons (* *p* < 0.05; ** *p* < 0.01, *** *p* < 0.005, **** *p* < 0.001). 

## Data Availability

Not applicable.

## Supplementary Materials

The following supporting information can be downloaded at: https://www.mdpi.com/article/10.3390/ijms23042016/s1.
