# Supplementary material for: Co-Administration of Simvastatin Does Not Potentiate the Benefit of Gene Therapy in the mdx Mouse Model for Duchenne Muscular Dystrophy
Source: Int J Mol Sci. 2022 Feb 11;23(4):2016. doi: 10.3390/ijms23042016 (PMC8878028; doi:10.3390/ijms23042016)
Supplement: Supplementary file 1 [file ijms-23-02016-s001.zip › ijms-1522969-supplementary.pdf]

International Journal of
Molecular Sciences

MDPI

Supplemental Figures

Fig-Sup-1a.
Microdystrophin VCN GA

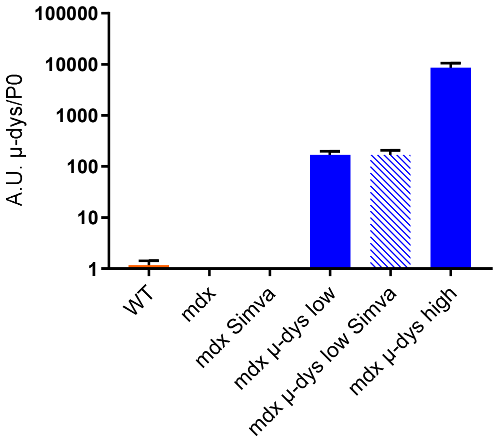

Fig-Sup-1b.
Microdystrophin VCN Diaphragm

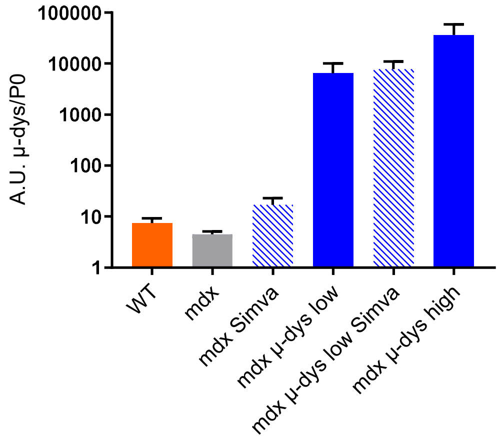

Fig-Sup-1c.
Microdystrophin mRNA GA muscle

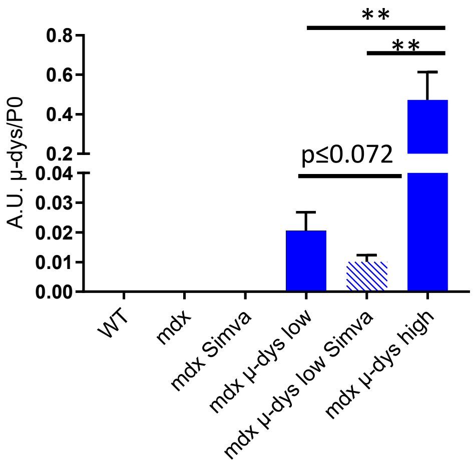

Fig-Sup-1d.
Microdystrophin mRNA Diaphragm

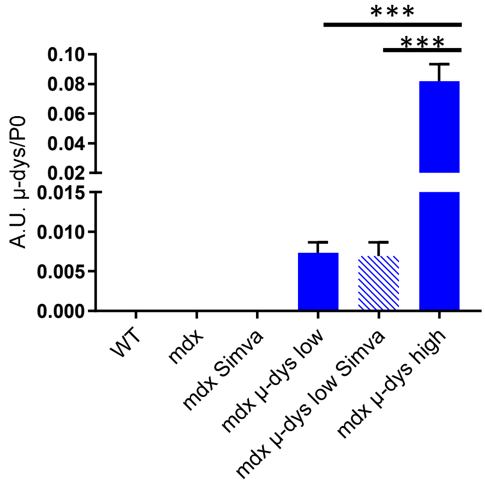

Fig-Sup-1e.
Microdystrophin protein GA muscle

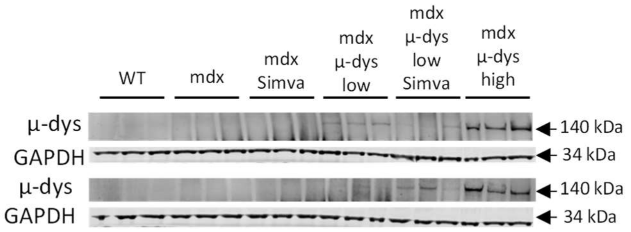

Fig-Sup-1f.
Quantification microdystrophin GA muscle

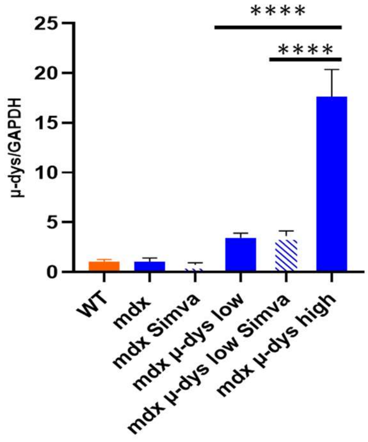

Fig-Sup-1g.
Microdystrophin positive fibers GA muscle

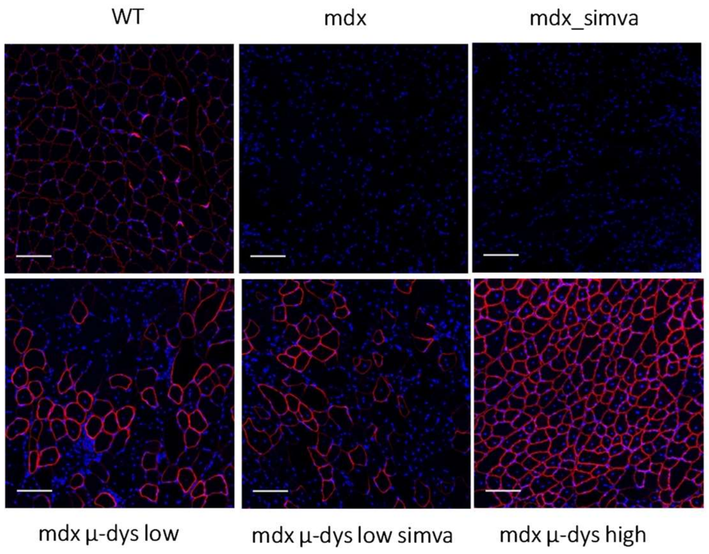

Fig-Sup-1h.
Microdystrophin positive fibers
(%) GA muscle

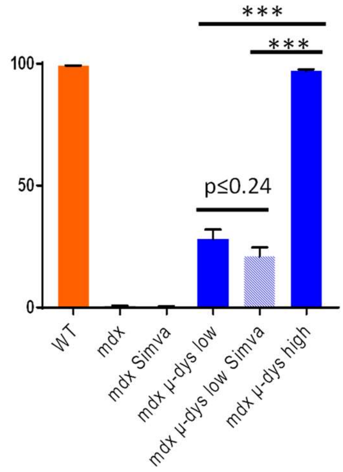

Supplemental Figure S1. Transgene expression. **(Sup-1a, 1b).** Vector copy number (VCN), normalized to the genomic *Rplp0* (P0) was evaluated in the GA (1a), and diaphragm (1b) muscles. **(Sup-1c, 1d).** mRNA expression of the microdystrophin (µ-dys) in the GA (1c), and diaphragm (1d) muscles was quantified by RT-qPCR. **(Supp-1e, f).** The expression of the microdystrophin in the GA muscles was quantified by a Western blotting (1e), and presented graphically in (1f) after normalization to GAPDH. **(Supp-1g, h).** Transversal sections of the GA muscles were stained for dystrophin expression (1g, scale bar = 100µm), and presented graphically in 1h.
